# Dynamical analysis of a stochastic delayed SIR epidemic model with vertical transmission and vaccination

**DOI:** 10.1186/s13662-022-03707-7

**Published:** 2022-04-22

**Authors:** Xiaolei Zhang, Maoxing Liu

**Affiliations:** Department of Mathematics, North University of China, Taiyuan, Shanxi 030051 P.R. China

**Keywords:** Stochastic delayed SIR epidemic model, Temporary immunity, Vaccination, Persistence, Extinction, Threshold

## Abstract

In order to describe the dynamic process of epidemic transmission with vertical transmission and vaccination in more detail and to better track the factors that lead to the occurrence of epidemics, we construct a stochastic delayed model with a specific functional response to describe its epidemic dynamics. We first prove the existence and uniqueness of the positive solution of the model. Moreover, we analyze the sufficient conditions for the extinction and persistence of the model. Finally, numerical simulations are presented to illustrate our mathematical findings.

## Introduction

A mathematical model has always been an important tool in the study of infectious diseases; there are many works about epidemic such as SIS, SIR, SEIR, and so on. In recent years, research on infectious diseases has been developing, and some good results have been achieved. Elaiw and Agha considered the delayed partial differential equation model to analyze Oncolytic virotherapy based on previous work about ODE [1]. In [2, 3], they all applied different models to analyze the SARS-CoV-2 and some useful suggestions were put forward. There are many other papers about epidemic model, such as [4–6]. All of these works show that it is an effective method to analyze infectious diseases using the infectious disease model.

**Figure 1:**
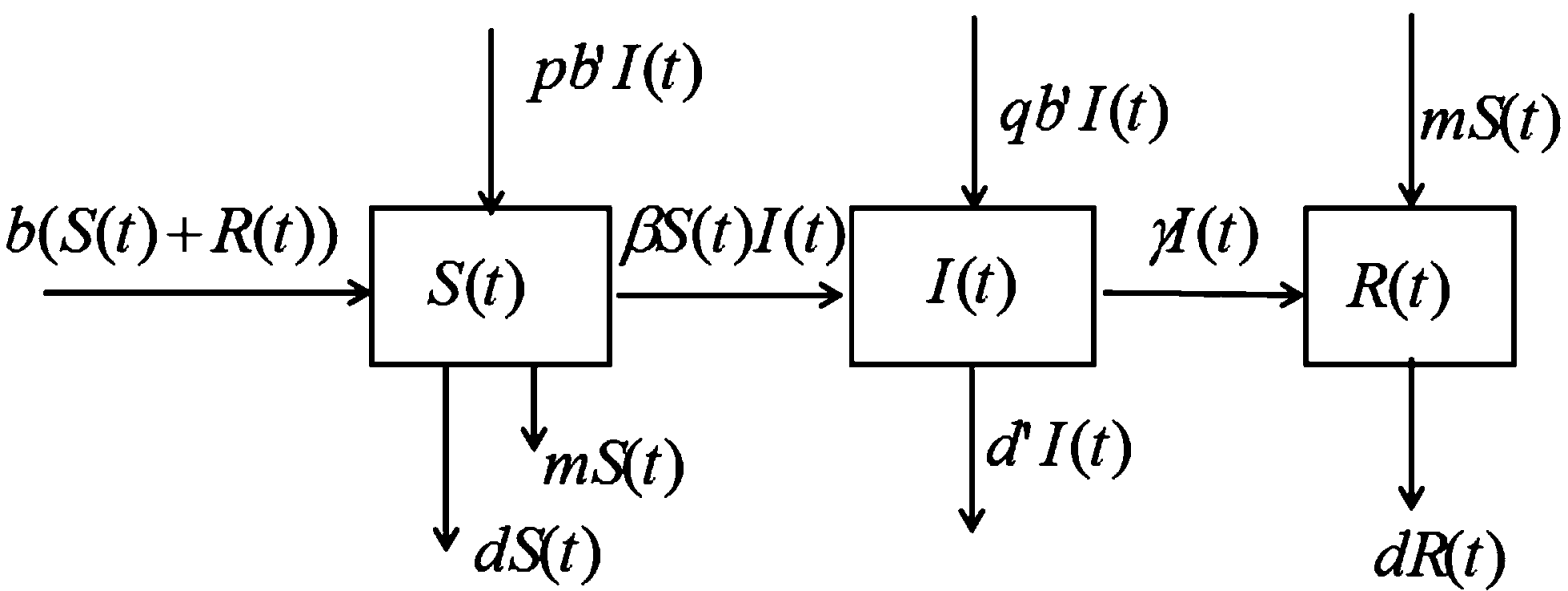
The compartmental diagram for the model

However, in real life, some infectious diseases may be transmitted vertically from one person to another; that is, the offspring of infected parents may be infected with infectious diseases such as hepatitis and tuberculosis at birth, called vertical transmission [7], so how to effectively prevent and control the spread of infectious diseases has become an important topic in epidemiology, on the current research shows that vaccination has become an important and commonly used strategy to eliminate infectious diseases, it can effectively reduce the infection of infectious diseases [8–10]. Based on the SIR epidemic model with vaccination and vertical transmission model proposed by Meng and Chen [11], we establish the following model, and a framework diagram of the disease model is shown in Fig. 1.

$$ \textstyle\begin{cases} \frac{{dS(t)}}{{dt}}=-\beta S(t)I(t)-dS(t)+pb'I(t)+b(S(t)+R(t))-mS(t), \\ \frac{{dI(t)}}{{dt}}=\beta S(t)I(t)-d'I(t)-\gamma I(t)+qb'I(t), \\ \frac{{dR(t)}}{{dt}}=\gamma I(t)-dR(t)+mS(t). \end{cases} $$

In the above model, $S(t)$, $I(t)$, and $R(t)$ denote the number of susceptible, infective, recovered individuals at time *t*, respectively. We suppose that *b* and $b'$ are the birth rate coefficients of the non-infected person $(S+R)$ and infected person; *I*, *d*, and $d'$ are their corresponding death coefficients, respectively. The infection rate of the disease is *β*; the susceptible person is an infected as infected person at a bilinear rate of $\beta I(t)$, and the infection recovery rate is *γ*. The proportion of the offspring of infectious parents who are susceptible is *p*; the proportion of the offspring of infectious parents who are infected is *q*, $0< p<1$, $0< q<1$ and $p+q=1$. *m* ($0< m<1$) is the proportion of the successfully vaccinated population to the entire susceptible population.

But in fact, the vaccine validity is usually limited, and the immunized person’s immunity can disappear [12–16]. Suppose *ω* denotes the period of vaccine validity, then the susceptible inoculated at $t-\omega $ will become susceptible again at *t*. However, due to the existence of natural mortality, the probability of these vaccinated people still alive at *t* is $e^{-d\omega }$ [17]. Then, we can get the model as follows:

1.1 $$ \textstyle\begin{cases} \frac{{dS(t)}}{{dt}} =-\beta S(t)I(t)-dS(t)+pb'I(t)+b\bigl(S(t)+R(t)\bigr)-mS(t)+mS(t- \omega )e^{-d\omega }, \\ \frac{{dI(t)}}{{dt}} =\beta S(t)I(t)-d'I(t)-\gamma I(t)+qb'I(t), \\ \frac{{dR(t)}}{{dt}} =\gamma I(t)-dR(t)+mS(t)-mS(t-\omega )e^{-d \omega }. \end{cases} $$

Here, we assume *b*, *d*, $b'$, and $d'$ to be equal; the case can be seen in [18, 19]. It can be found from the model that $\frac{{d(S(t)+I(t)+R(t))}}{{dt}}=0$ and the population has a constant size, which is normalized to unity. Our analysis below is simplified with this assumption. By calculations, the basic reproduction number of model (1.1) is obtained $R_{0}^{1}=\frac{\beta b}{(b+m(1-e^{-b\omega }))(pb+\gamma )}$, and we find that when $R_{0}^{1}<1$, the model has a disease-free equilibrium point $(S_{0},0,R_{0})$, where $S_{0}=\frac{b}{b+m(1-e^{-b \omega })}$, $R_{0}=\frac{m(1-e^{-b \omega })}{b+m(1-e^{-b \omega })}$, when $R_{0}^{1}>1$, the model has an endemic equilibrium point $(S_{e},I_{e},R_{e})$, where $S_{e}=\frac{bp+\gamma }{\beta }$, $I_{e}=\frac{b}{b+r}(1-\frac{1}{R_{0}^{1}})$, $R_{e}= \frac{\beta \gamma R_{0}^{1}+\beta b-(bp+\gamma )(b+\gamma )R_{0}^{1}}{\beta R_{0}^{1}(b+\gamma )}$.

To model the disease transmission process, several authors improved the following bilinear incidence rate $\beta SI$ to get a more suitable infection rate, where *β* is a positive constant [20]. However, there are many forms of nonlinear incidence rates for a more generalized form. The incidence rate $\beta SI/(1 + \alpha _{1}S + \alpha _{2}I + \alpha _{3}SI)$ was introduced by Hattaf et al. [21] and used in [22]; it is a general form, which represents mutual interference between *S* and *I*, *βI* and measures the infectivity of the disease when it enters a fully susceptible population. $1/(1+\alpha _{1}S+ \alpha _{2}I + \alpha _{3}SI)$ measures the inhibition effect from the behavioral change of the susceptible population and the infected population when their number increases or from the crowding effect of the infected individuals, that is, due to the information of the disease. The infected or susceptible population will cause behavioral changes and inhibitory effects; therefore, it is more interesting and valuable than bilinear incidence rate. It has been widely applied in epidemiological studies. When $\alpha _{1} = \alpha _{3} = 0$, then we have the saturated incidence rate $\beta SI/(1+\alpha _{2}I)$ [23], which was used in [24–26]; when $\alpha _{3}=0$, we get the Beddington–DeAngelis functional response $\beta SI/(1+\alpha _{1}S + \alpha _{2}I)$ [27], which was used in [28, 29]; when $\alpha _{3}= \alpha _{1} \alpha _{2}$, we get the Crowley–Martin functional response $\beta SI/(1 + \alpha _{1}S + \alpha _{2}I + \alpha _{1}\alpha _{2}SI)$ [30]. $\alpha _{1}$, $\alpha _{2} $, and $\alpha _{3}$ are used to measure the inhibitory effect on infectious diseases when crowding effect or behavioral changes caused by the increase of susceptible individuals occur, and the infection coefficient can be effectively reduced by reasonably selecting appropriate parameters.

In this article, a Crowley–Martin functional response is considered; that is, the incidence rate of disease is modeled by $\beta SI/f(S,I)=\beta SI/(1+\alpha _{1} S+\alpha _{2} I+\alpha _{1} \alpha _{2}S I)$, where *β* is the infection coefficient and $\alpha _{1}$, $\alpha _{2}\geq 0$ are constants. Thus, we get

1.2 $$ \textstyle\begin{cases} \frac{{dS(t)}}{{dt}} =- \frac{\beta S(t)I(t)}{f(S,I)}-bS(t)+pbI(t)+b(1-I(t))-mS(t)+mS(t- \omega )e^{-b\omega }, \\ \frac{{dI(t)}}{{dt}} =\frac{\beta S(t)I(t)}{f(S,I)}-\gamma I(t)-pbI(t), \\ \frac{{dR(t)}}{{dt}} =\gamma I(t)-bR(t)+mS(t)-mS(t-\omega )e^{-b \omega }. \end{cases} $$

For model (1.2), we can use the results presented by Hattaf et al. [31]. It is easy to get the basic reproduction number of disease that is given by

1.3 $$ R_{0}^{2}=\frac{f(S_{0},0)}{pb+\gamma }= \frac{\beta b}{(b+\alpha _{1}b+m(1-e^{-b\omega }))(pb+\gamma )}.$$

On the other hand, the current environmental fluctuations have a great impact on all aspects of real life, so we will consider the impact of environmental fluctuations on the transmission rate *β*. Unless otherwise specified, it is assumed here that the random disturbance is a type of white noise, namely $\beta \,dt\rightarrow \beta \,dt+\sigma \, dB(t)$, where $B(t)$ is a Brownian motion, and *σ* is intensity. We let $B(t)$ be defined on a complete probability space $(\Omega ,\mathcalligra{F},\{\mathcalligra{F}_{t}\}_{t\geq 0},P)$ with a filtration $\{\mathcalligra{F}_{t}\}$ satisfying conditions that are increasing and right continuous while $\mathcalligra{F}_{0}$ contains all P-null sets. Then the form of the stochastic model corresponding to the deterministic model (1.2) is as follows

1.4 $$ \textstyle\begin{cases} dS(t)= [- \frac{\beta S(t)I(t)}{f(S,I)}-bS(t)+pbI(t)+b(1-I(t))-mS(t)+mS(t- \omega )e^{-b\omega } ] \,dt \\ \hphantom{dS(t)={}}{} -\frac{\sigma SI}{f(S,I)}\,dB(t), \\ dI(t)= [\frac{\beta S(t)I(t)}{f(S,I)}-\gamma I(t)-pbI(t) ]\,dt+ \frac{\sigma SI}{f(S,I)}\,dB(t), \\ dR(t)= [\gamma I(t)-bR(t)+mS(t)-mS(t-\omega )e^{-b\omega } ]\,dt. \end{cases} $$

For biological significance, the following analysis satisfies the condition $S(t)\geq 0$, $I(t)\geq 0$, $R(t)\geq 0$. Then, noticing the first two stochastic differential equations in system (1.4) do not depend on the function $R(t)$, we can exclude the third one without loss of generality [32, 33]. Hence, we will only discuss the following system:

1.5 $$ \textstyle\begin{cases} dS(t)= [- \frac{\beta S(t)I(t)}{f(S,I)}-bS(t)+pbI(t)+b (1-I(t) )-mS(t)+mS(t- \omega )e^{-b\omega } ]\,dt \\ \hphantom{dS(t)={}}{} -\frac{\sigma SI}{f(S,I)}\,dB(t), \\ dI(t)= [\frac{\beta S(t)I(t)}{f(S,I)}-\gamma I(t)-pbI(t) ]\,dt+ \frac{\sigma SI}{f(S,I)}\,dB(t). \end{cases} $$

This paper is organized as follows: in Sect. 2, the global existence, positivity, and boundedness of solutions of our stochastic model (1.5) will be proved. In Sects. 3 and 4, we respectively show sufficient conditions for the extinction and persistence of the disease. In Sect. 5, some numerical simulations are presented to illustrate our main results. Finally, the paper ends with a brief discussion and conclusion in Sect. 6.

## Existence of the positive solution

In this section, we establish the global existence, positivity, and boundedness of solutions of system (1.5). Since $S(t)$ and $I(t)$ in system (1.5) denote population sizes, they should be nonnegative, so for further study, we should firstly give region to prove that system (1.5) has a unique global positive solution. First, we can find that it is clear that region

$$ \Delta = \biggl\{ (S,I)\in R^{2}_{+}:S+I\leq { \frac{b}{b+m(1-e^{-b\omega })}} \biggr\} , $$

is a positive invariant set of the deterministic model (1.2). Here, we will show that the region Δ is almost surely a positive invariant set of the corresponding stochastic model (1.5), i.e., if $X_{0} = (S(0), I(0))\in \Delta $, then $P(X(t)\in \Delta )=1$ for all $t \geq 0$ [34].

### Theorem 2.1

*The region* Δ *is almost surely positive invariant of stochastic model* (1.5).

### Proof

Let $(S(\theta ), I(0))\in \Delta $, $\theta \in [-\omega , 0)$ and $n_{0} > 0$ be sufficiently large such that each component of $(S(\theta ), I(0))$ is contained within the interval $( \frac{1}{n_{0}},{\frac{b}{b+m(1-e^{-b\omega })}} ]$. Define, for each integer $n \geq n_{0}$, the stopping times

$$\begin{aligned}& \tau _{n}=\inf \biggl\{ {t>0:X(t)\in \Delta \text{ and } X(t) \in \biggl( \frac{1}{n},\frac{b}{b+m(1-e^{-b\omega })} \biggr) } \biggr\} ,\\& \tau =\inf \bigl\{ {t>0:X(t)\notin \Delta } \bigr\} . \end{aligned}$$

It suffices to prove that $P(\tau =\infty )=1$, that is $P(\tau < t)=0$, $\forall t > 0$, we can see clearly that $P(\tau < t) \leq P(\tau _{n} < t)$. We only need to show that $\limsup_{n \to \infty } P(\tau _{n}< t) =0$ for this matter, referring to [35, 36], we can take a similar function and use some approaches to prove the theorem. Then, we set a $C^{2}$-function *U*: $R^{2}_{+}\rightarrow R_{+}$ for all $(S(t), I(t))> 0$:

$$ U(X)=\frac{1}{S}+\frac{1}{I}. $$

Applying Itô’s formula, for all $t\geq 0$ and $s \in [0, t \wedge \tau _{n}]$, we obtain

$$\begin{aligned}& d{U}\bigl(X(s)\bigr) \\& \quad = \biggl[ \biggl( \frac{\beta I}{Sf(S,I)}+ \frac{b}{S}- \frac{pbI}{S^{2}}-\frac{b(1-I)}{S^{2}}+ \frac{m}{S} - \frac{me^{-b\omega }S(t-\omega )}{S^{2}}+\frac{1}{S} \biggl( \frac{\sigma I}{f(S,I)} \biggr)^{2} \biggr) \biggr]\,ds \\& \qquad {}+ \biggl[{ \biggl( -\frac{\beta S}{If(S,I)}+\frac{pb+\gamma }{I}+ \frac{1}{I} \biggl(\frac{\sigma S}{f(S,I)} \biggr)^{2} \biggr)} \biggr]\,ds + \biggl[{\biggl( \frac{\sigma (I^{2}-S^{2})}{SIf(S,I)} \biggr)} \biggr]\,dB(t) \\& \quad \leq \bigl[\beta I+b+m+(\sigma I)^{2} \bigr]\frac{ds}{S}+ \bigl[ pb+ \gamma +(\sigma S)^{2} \bigr]\frac{ds}{I}+ \biggl[ \frac{\sigma (I^{2}-S^{2})}{SIf(S,I)} \biggr] \,dB(t) \\& \quad \leq \bigl[\beta +b+m+\sigma ^{2} \bigr] \frac{ds}{S}+ \bigl[pb+ \gamma +\sigma ^{2} \bigr] \frac{ds}{I}+ \biggl[ \frac{\sigma (I^{2}-S^{2})}{SIf(S,I)} \biggr] \,dB(t). \end{aligned}$$

Then

2.1 $$ d{U}\bigl(X(s)\bigr)\leq \eta {U}\bigl(X(s) \bigr)\,ds+ \biggl[ \frac{\sigma (I^{2}-S^{2})}{SIf(S,I)} \biggr] \,dB(t), $$

where $\eta =\max \{ \beta +b+m+\sigma ^{2}, pb+\gamma +\sigma ^{2} \}$. Taking integral and expectations on both sides of (2.1) and applying Fubini’s theorem, we get

$$ {\mathrm{E}} {U}\bigl(X(s)\bigr)\leq {U}(X_{0})+\eta \int ^{s}_{0} {\mathrm{E}} {U}\bigl(X(u) \bigr)\,du.$$

Using Gronwall’s inequality, we have

$$ \forall s\in [0,t\wedge \tau _{n}],\quad {\mathrm{E}} {U}\bigl(X(s) \bigr)\leq {U}(X_{0})e^{ \eta s}.$$

Hence,

2.2 $$ {\mathrm{E}} {U}\bigl(X(t\wedge \tau _{n})\bigr)\leq {U}(X_{0})e^{\eta (t\wedge \tau _{n})}\leq {U}(X_{0})e^{\eta t},\quad \forall t\geq 0. $$

Since ${U}(X(t\wedge \tau _{n}))>0$ and some component of $X(\tau _{n})$ is less than or equal to $\frac{1}{n}$, we deduce that

2.3 $$ {\mathrm{E}} {U}\bigl(X(t\wedge \tau _{n})\bigr)\geq {\mathrm{E}} {U}\bigl[X(\tau _{n})_{X\{ \tau _{n}< t\}} \bigr]\geq nP(\tau _{n}< t). $$

By (2.2) and (2.3), we get for all $t\geq 0$

$$ P(\tau _{n}< t)\leq \frac{{U}(X_{0})e^{\eta t}}{n}.$$

Thus, $\limsup_{n \to \infty } P(\tau _{n}< t) =0$. This completes the proof. □

The following theorem proves that there is a unique globally positive solution to system (1.5) for any initial value $X_{0}=(S(\theta ),I(0))\in R_{+}^{2}$, where $R_{+}^{2}=\{({X_{1},X_{2}})\in R^{2}\mid :X_{i}>0,i=1,2\}$.

### Theorem 2.2

*For any initial value* $S(\theta )\geq 0$ *and* $I(0)>0$, $\forall \theta \in [-\omega , 0)$, *system* (1.5) *has a unique positive solution* $(S(t), I(t))$ *on* $t > 0$, *and the solution will remain in* $R^{2}_{+}$ *with probability one*, *that is to say* $(S(t),I(t))\in R^{2}_{+}$ *for all* $t > 0$ *almost surely*.

### Proof

Since the coefficients of system (1.5) satisfy the local Lipschitz conditions, then for any initial value $S(\theta )\geq 0$ for all $\theta \in [-\omega , 0)$ and $S(0)>0$, $I(0)>0$, there is a unique local solution $(S(t), I(t))$ on $t\in [0, \tau _{e})$, where $\tau _{e}$ represents the explosion time [37]. To verify this solution is global, we only need to show $\tau _{e}=\infty $ a.s. To this end, let $k_{0} \geq 1$ be sufficiently large such that $(S(\theta ),I(0))$ belongs to the interval $[\frac{1}{k_{0}} , k_{0}]$. For each integer $k \geq k_{0}$, let us define the following stopping time

$$ \tau _{k}=\inf \biggl\{ {t\in [{0,{\tau _{e}}}]:S(t) \notin \biggl( \frac{1}{k} , k \biggr) \text{ or } I(t)\notin \biggl(\frac{1}{k} , k \biggr) } \biggr\} ,$$

where throughout this paper, we set $\inf \emptyset =\infty $ (as usual, ∅ represents the empty set). Obviously, $\tau _{k}$ is increasing as $k\rightarrow \infty $. Let $\tau _{\infty }= \lim_{k \to \infty }\tau _{k}$, whence $\tau _{\infty }\leq \tau _{e}$ a.s. If $\tau _{\infty }=\infty $ a.s. is true, then $\tau _{e}=\infty $ a.s. and $(S(t), I(t))\in R^{2}_{+}$ a.s. for all $t > 0$. That is to say, to complete the proof, we only need to show $\tau _{\infty }=\infty $ a.s. If this assertion is false, then there exists a pair of constants $T > 0$ and $\epsilon \in (0, 1)$ such that $P\{\tau _{\infty }\leq T\}>\epsilon $. Thereby, there is an integer $k_{1}\geq k_{0}$ such that

2.4 $$ P\{\tau _{k}\leq T\}\geq \epsilon\quad \text{for all } k\geq k_{1}. $$

Define

$$ {V}(S,I)=(S-1-\ln S)+(I-1-\ln I)+me^{-b\omega }\int ^{t}_{t-\omega }S(s)\,ds, $$

the nonnegativity of the above function can be seen from $u-1-\ln u \geq 0 $ for $\forall u > 0$, let $k \geq k_{0} $ and $T > 0$ be arbitrary. Applying Itô’s formula, for all $t\ge 0$, we can get:

$$ d{V}(S,I)=LV(S,I)\,dt+{\sigma }({I-S})\, d{B(t)}, $$

where

$$\begin{aligned} LV(S,I) =&-\frac{\beta SI}{f(S,I)}-bS+pbI+b(1-I)+me^{-b\omega }S(t- \omega )+ \frac{\beta I}{f(S,I)}+b-\frac{pbI}{S} \\ &{}-\frac{b(1-I)}{S}+m-\frac{me^{-b\omega }S(t-\omega )}{S}+ \frac{\beta SI}{f(S,I)}-(pb+r)I- \frac{\beta S}{f(S,I)}+pb+\gamma \\ &{}-m\bigl(1-e^{-b\omega }\bigr)S-me^{-b\omega }S(t-\omega )+ { \frac{1}{2} \biggl( \frac{\sigma I}{f(S,I)} \biggr)^{2}+ \frac{1}{2} \biggl( \frac{\sigma S}{f(S,I)} \biggr)^{2}} \\ \leq & \frac{\beta I}{f(S,I)}+2b+m+pb+\gamma +\frac{1}{2} \biggl( \biggl(\frac{\sigma I}{f(S,I)} \biggr)^{2}+ \biggl( \frac{\sigma S}{f(S,I)} \biggr)^{2} \biggr) , \end{aligned}$$

here because $f(S,I)\geq 0$, so $LV(S,I)\leq {\beta +2b+m+pb+\gamma +\sigma ^{2}}=:K$, where $K > 0$ is a constant. So, the above can be written as

2.5 $$ d{V}(S,I)\leq { K }\,dt+{\sigma }({I-S})\,d{B(t)}, $$

integrating the above inequality (2.5), we obtain:

$$ {V}(S,I)\leq {V}( {S( 0 ),I( 0 )}) + Kt+\int ^{t}_{0}{{\sigma }( {I-S})\,d{B}} $$

because $\int _{0}^{t}{{\sigma }({I-S})\,d{B}}$. It is a process with a mean value of 0, taking expectation, we obtain

$$ {\mathrm{E}}V \bigl( {S( {{\tau _{k}} \wedge T} ),I( {{\tau _{k}} \wedge T} )} \bigr) \leq {V}\bigl( {S(0),I(0)}\bigr) + K{ \mathrm{E}}({{\tau _{k}} \wedge T}). $$

Thus

2.6 $$ {\mathrm{E}}V \bigl( {S( {{\tau _{k}} \wedge T} ),I( {{\tau _{k}} \wedge T} )} \bigr)\leq {V}\bigl({S(0),I(0)}\bigr) + K T. $$

Set $\Omega _{k}=\{\tau _{k} \leq T\}$ for $k\geq k_{1}$ and by virtue of (2.4), we obtain $P(\Omega _{k})\geq \epsilon $. Note that for every $\omega \in \Omega _{k}$, the $S(\tau _{k}, \omega )$ or $I(\tau _{k},\omega )$ equals either *k* or $\frac{1}{k}$. Consequently, $V(S(\tau _{k}, \omega ),I(\tau _{k}, \omega ))$ is no less than either $k-1-\ln k$ or $\frac{1}{k}-1-\ln {\frac{1}{k}}=\frac{1}{k}-1+\ln k$. Hence, we can get

$$ {V} \bigl(S(\tau _{k}, \omega ), I(\tau _{k}, \omega ) \bigr)\geq [k-1- \ln k]\wedge \biggl[\frac{1}{k}-1+\ln {k} \biggr].$$

It follows from (2.6) that

$$\begin{aligned} {V}\bigl({S(0),I(0)}\bigr) + K T \geq &{\mathrm{E}}\bigl[I_{\Omega _{k}}( \omega )V\bigl(S( \tau _{k}, \omega ), I(\tau _{k}, \omega )\bigr)\bigr] \\ \geq &\epsilon \biggl[(k-1-\ln k)\wedge \biggl(\frac{1}{k}-1+\ln {k} \biggr) \biggr], \end{aligned}$$

where $I_{\Omega _{k}}$ is the indicator function of $\Omega _{k}$. Letting $k \rightarrow \infty $, then we have $\infty >{V}({S(0),I(0)})+K T=\infty $, which yields the contradiction, we get $\tau _{\infty }=\infty $. This means that $S(t)$ and $I(t)$ will not explode in a finite time, almost surely. This completes the proof. □

## Extinction of the disease

In this section, we study the extinction of the disease. Before giving our main result of this section, let us present some lemmas.

### Lemma 3.1

*Let* $(S(t), I(t))$ *be the solution of system* (1.5) *with any initial value* $I(0) > 0$ *and* $S(\theta )\geq 0$ *for all* $\theta \in [-\omega , 0)$ *with* $S(0) > 0$, *then*

$$ \lim_{t \to \infty }{ \frac{S(t)+I(t)+me^{-bt}\int ^{t}_{t-\omega }e^{bs}S(s)\,ds}{t}}=0 \quad \textit{a.s.}$$

*Furthermore*,

$$ \lim_{t \to \infty }\frac{S(t)}{t}=0, \qquad \lim_{t \to \infty } \frac{I(t)}{t}=0,\qquad \lim_{t \to \infty } \frac{e^{-bt}\int ^{t}_{t-\omega }e^{bs}S(s)\,ds}{t}{t}=0 \quad \textit{a.s.} $$

### Proof

The proof is similar to that in Liu et al. [38] and hence is omitted. □

### Lemma 3.2

*Let* $M = \{M_{t}\}_{t\geq 0}$ *be a real*-*valued continuous local martingale vanishing at* $t = 0$. *Then*

$$ \lim_{t \to \infty }{\langle M,M\rangle }_{t}= \infty\quad \textit{a.s.} \quad \Rightarrow \quad \lim_{t \to \infty }\frac{M_{t}}{ {\langle M,M\rangle }_{t}} =0 \quad \textit{a.s.} $$

*and also*

$$ \limsup_{t \to \infty } \frac{ {\langle M,M\rangle }_{t} }{t}< \infty \quad \textit{a.s.} \quad \Rightarrow \quad \lim_{t \to \infty }\frac{M_{t}}{t} =0\quad \textit{a.s.}$$

Now we will give our main result of this section.

### Theorem 3.1

*Let* $(S(t), I(t))$ *be the solution of system* (1.5) *with initial value* $(S(\theta ), I(0))\in \Delta $, $\theta \in [-\omega ,0)$, *assume that* (a) $\sigma ^{2}>\frac{\beta ^{2}}{2(pb+\gamma )}$, (b) $R_{0}^{s}<1$ *and* $\sigma ^{2}\leq \frac{\beta (b+\alpha _{1}b+m(1-e^{-b\omega }))}{b}$. *Then*

- *if* (a) *holds*,
  3.1 $$ \limsup_{t \to \infty } \frac{\ln I(t)}{t}\leq \frac{\beta ^{2}}{2\sigma ^{2}}-(pb+\gamma )< 0 \quad \textit{a.s.}, $$
- *if* (b) *holds*,
  3.2 $$ \limsup_{t \to \infty } \frac{\ln I(t)}{t}\leq \bigl(R_{0}^{s}-1\bigr) (pb+ \gamma )< 0 \quad \textit{a.s.}, $$

*where*

$$ R_{0}^{s}=R_{0}^{2} \biggl(1- \frac{\sigma ^{2}b}{2\beta (b+\alpha _{1}b+m(1-e^{-b\omega }))} \biggr), $$

*namely*, $I(t)$ *tends to zero exponentially a*.*s*., *the disease fades with probability* 1.

### Proof

It follows from Itô’s formula that

$$ d\ln I= \biggl[\frac{\beta S}{f(S,I)}-(pb+\gamma )-\frac{1}{2} \biggl( \frac{\sigma S}{f(S,I)} \biggr)^{2} \biggr]\,dt+\frac{\sigma S}{f(S,I)}\,dB, $$

integrating this from 0 to *t* and dividing by *t* on both sides, we have

3.3 $$ \frac{\ln I(t)}{t}=\frac{1}{t} \int ^{t}_{0} \biggl[ \frac{\beta S(s)}{f(S,I)}-(pb+ \gamma )-\frac{1}{2} \frac{\sigma ^{2} S^{2}(s)}{f^{2}(S,I)} \biggr]\,ds+ \frac{\ln I(0)}{t}+ \frac{M(t)}{t}, $$

where $M(t)=\int ^{t}_{0} \sigma \frac{S(s)}{f(S(s),I(s))} \,dB(s)$. By the large number theorem for martingales (Lemma 3.2) [39], we have

$$ \lim_{t \to \infty }\frac{M_{t}}{t}= \lim_{t \to \infty } \frac{B(t)}{t}=0 \quad \mbox{a.s.}, $$

if the condition (a) is satisfied, Eq. (3.3) becomes

$$\begin{aligned} \frac{\ln I(t)}{t} =&\frac{1}{t} \int ^{t}_{0} \biggl[-\frac{1}{2} \sigma ^{2} \biggl(\frac{S(s)}{f(S,I)}-\frac{\beta ^{2}}{\sigma ^{2}} \biggr)^{2}-(pb+\gamma )+\frac{\beta ^{2}}{2\sigma ^{2}} \biggr]\,ds+ \frac{\ln I(0)}{t}+\frac{M(t)}{t} \\ \leq & \biggl[-(pb+\gamma )+\frac{\beta ^{2}}{2\sigma ^{2}} \biggr]+ \frac{\ln I(0)}{t}+\frac{M(t)}{t}, \end{aligned}$$

taking the limit superior of both sides, we obtain the desired assertion (3.1).

On the other hand, noting that the Crowley–Martin functional response can be written differently as

3.4 $$ \begin{aligned} \frac{\beta S}{f(S,I)}&= \frac{\beta S}{1+\alpha _{1}S+\alpha _{2}I+\alpha _{1}\alpha _{2}SI} \\ &= \frac{\beta b}{b+\alpha _{1}b+m(1-e^{-b\omega })} \\ &\quad {}- \frac{\beta (b+m(1-e^{-b\omega }) )}{(b+\alpha _{1}b+m(1-e^{-b\omega }))(1+\alpha _{1}S+\alpha _{2}I+\alpha _{1}a_{2}SI)} \\ &\quad {}\times\biggl(\frac{b}{b+m(1-e^{-b\omega })}-S \biggr) \\ &\quad {}- \frac{\beta b\alpha _{2}}{ (b+\alpha _{1}b+m(1-e^{-b\omega }) )(1+\alpha _{1}S+\alpha _{2}I+\alpha _{1}\alpha _{2}SI)}I \\ &\quad {}- \frac{\beta b\alpha _{1}\alpha _{2}}{ (b+\alpha _{1}b+m(1-e^{-b\omega }) )(1+\alpha _{1}S+\alpha _{2}I+\alpha _{1}\alpha _{2}SI)}SI, \end{aligned} $$

thus, if (b) holds, then we can transpose (3.3) into

$$\begin{aligned} \frac{\ln I(t)}{t} \leq& \frac{\beta b}{b+\alpha _{1}b+m(1-e^{-b\omega })}-(pb+\gamma )- \frac{1}{2} \biggl(\frac{\sigma b}{b+\alpha _{1}b+m(1-e^{-b\omega })} \biggr)^{2} \\ &{}+ \frac{\ln I(0)}{t}+\frac{M(t)}{t}. \end{aligned}$$

By the law of large number for martingales and for $R_{0}^{s} < 1$, we obtain

$$\begin{aligned} \frac{\ln I(t)}{t} \leq & \biggl[R_{0}^{2} \biggl(1- \frac{\sigma ^{2}b}{2\beta (b+\alpha _{1}b+m(1-e^{-b\omega }))} \biggr)-1 \biggr](pb+\gamma )+\frac{\ln I(0)}{t}+ \frac{M(t)}{t} \\ \leq & \bigl(R_{0}^{s}-1\bigr) (pb+\gamma )+ \frac{\ln I(0)}{t}+\frac{M(t)}{t}, \end{aligned}$$

taking the limit superior of both sides, we get the assertion (3.2). We have proved that

$$ \limsup_{t \to \infty }\frac{\ln I(t)}{t}\leq \lambda _{I}< 0 \quad \mbox{a.s.},$$

where $\lambda _{I}=\frac{\beta ^{2}}{2\sigma ^{2}}-(pb+\gamma )$ if $(a)$ holds; $\lambda _{I}=(R_{0}^{s}-1)(pb+\gamma )$ if (b) holds. This completes the proof. □

## Persistence of the disease

In this section, we study the conditions for the Persistence of the disease. For simplicity, we define $\langle X(t)\rangle =\frac{1}{t}\int ^{t}_{0} X(s)\,ds$ and present the definition of persistence in the mean as follows [37].

### Definition 4.1

System (1.5) is said to be persistent in the mean if

$$ \liminf_{t \to \infty }\bigl\langle S(t)\bigr\rangle >0,\qquad \liminf _{t \to \infty }\bigl\langle I(t)\bigr\rangle >0\quad \mbox{a.s.}$$

### Lemma 4.1

*Let* $g\in C ([0,\infty )\times \Omega ,(0,\infty ) )$ *and* $G\in C([0,\infty )\times \Omega ,R)$. *If there exist two real numbers* $\lambda _{0}\geq 0$ *and* $\lambda > 0$ *for all* $t\geq 0$ *such that*

$$ \ln g(t)\geq \lambda _{0}t-\lambda \int _{0}^{t}g(s)\,ds+G(t)\quad \textit{and}\quad \lim_{t \to \infty } \frac{G(t)}{t}=0\quad \textit{a.s.}, $$

*then*

$$ \liminf_{t \to \infty }\bigl\langle g(t)\bigr\rangle \geq \frac{\lambda _{0}}{\lambda } \quad \textit{a.s.} $$

### Theorem 4.1

*Suppose that* $R_{0}^{s} >1$, *then the solution* $(S(t),I (t))$ *to system* (1.5) *is persistent in the mean for any given initial value* $(S(\theta ),I (0))\in \Delta $, $\theta \in [-\omega ,0)$. *Moreover*,

4.1 $$\begin{aligned}& \liminf_{t \to \infty } \bigl\langle I(t) \bigr\rangle \geq I_{*}>0, \end{aligned}$$

4.2 $$\begin{aligned}& \liminf_{t \to \infty } \biggl\langle \frac{b}{b+m(1-e^{-b\omega })}-S(t) \biggr\rangle \geq \frac{b+\gamma }{b+m(1-e^{-b\omega })}I_{*}>0, \end{aligned}$$

*where*

$$ I_{*}= \frac{(pb+\gamma )(R_{0}^{s}-1) (b+\alpha _{1}b+m(1-e^{-b\omega }) ) (b+m(1-e^{-b\omega }) )}{\beta [(b+\gamma ) (b+m(1-e^{-b\omega }) )+\alpha _{2}b (b+m(1-e^{-b\omega })+\alpha _{1}\alpha _{2}b^{2} ) ]}.$$

### Proof

Since $(S(t),I(t))\in \Delta $, from the Crowley–Martin functional response (3.4), we get

$$\begin{aligned} \frac{\beta S}{f(S,I)}&\geq \frac{\beta b}{b+\alpha _{1}b+m(1-e^{-b\omega })} \\ &\quad {}- \frac{\beta (b+m(1-e^{-b\omega }) )}{ (b+\alpha _{1}b+m(1-e^{-b\omega }) )} \biggl(\frac{b}{b+m(1-e^{-b\omega })}-S \biggr) \\ &\quad {}- \frac{\beta b\alpha _{2}}{ (b+\alpha _{1}b+m(1-e^{-b\omega }) )}I- \frac{\beta b^{2}\alpha _{1}\alpha _{2}}{ (b+\alpha _{1}b+m(1-e^{-b\omega }) ) (b+m(1-e^{-b\omega }) )}I \\ &\geq \frac{\beta (b+m(1-e^{-b\omega }) )}{ (b+\alpha _{1}b+m(1-e^{-b\omega }) )}S- \frac{\beta b\alpha _{2}}{ (b+\alpha _{1}b+m(1-e^{-b\omega }) )} \biggl(1+ \frac{b\alpha _{1}}{b+m(1-e^{-b\omega })} \biggr)I. \end{aligned}$$

We have $0\leq \frac{S}{f(S,I)}\leq \frac{b}{b+\alpha _{1}b+m(1-e^{-b\omega })}$, then

4.3 $$ \begin{aligned} d\ln I&= \biggl[\frac{\beta S}{f(S,I)}-(pb+\gamma )- \frac{1}{2} \biggl(\frac{\sigma S}{f(S,I)} \biggr)^{2} \biggr]\,dt+ \frac{\sigma S}{f(S,I)}\,dB \\ &\geq \biggl[\frac{\beta S}{f(S,I)}-(pb+\gamma )-\frac{1}{2} \biggl( \frac{\sigma b}{b+\alpha _{1}b+m(1-e^{-b\omega })} \biggr)^{2} \biggr]\,dt+ \frac{\sigma S}{f(S,I)}\,dB \\ &\geq \biggl[ \frac{\beta S (b+m(1-e^{-b\omega }) )}{b+\alpha _{1}b+m(1-e^{-b\omega })}-(pb+ \gamma )-\frac{1}{2} \biggl( \frac{\sigma b}{b+\alpha _{1}b+m(1-e^{-b\omega })} \biggr)^{2} \biggr]\,dt \\ &\quad {}- \biggl[ \frac{\beta b\alpha _{2}}{ (b+\alpha _{1}b+m(1-e^{-b\omega }) )} \biggl(1+\frac{b\alpha _{1}}{b+m(1-e^{-b\omega })} \biggr) \biggr]I\,dt+ \frac{\sigma S}{f(S,I)}\,dB, \end{aligned} $$

integrating both sides of (4.3) from 0 to *t*, there is

$$\begin{aligned} \ln I(t)-\ln I(0) \geq& \frac{\beta (b+m(1-e^{-b\omega }) )}{b+\alpha _{1}b+m(1-e^{-b\omega })} \int _{0}^{t}S(\theta )\,d\theta -(pb+\gamma )t \\ &{}-\frac{1}{2} \biggl( \frac{\sigma b}{b+\alpha _{1}b+m(1-e^{-b\omega })} \biggr)^{2}t \\ &{}- \biggl[ \frac{\beta b\alpha _{2}}{ (b+\alpha _{1}b+m(1-e^{-b\omega }) )} \biggl(1+\frac{b\alpha _{1}}{b+m(1-e^{-b\omega })} \biggr) \biggr] \\ &{}\times \int _{0}^{t} I(\theta )\,d\theta +M(t), \end{aligned}$$

then

4.4 $$ \begin{aligned} \ln I(t)&\geq \frac{\beta (b+m(1-e^{-b\omega }) )}{b+\alpha _{1}b+m(1-e^{-b\omega })} \int _{0}^{t}S(\theta )\,d\theta -(pb+\gamma )t \\ &\quad {}-\frac{1}{2} \biggl(\frac{\sigma b}{b+\alpha _{1}b+m(1-e^{-b\omega })} \biggr)^{2}t \\ &\quad {}- \biggl[ \frac{\beta b}{ (b+\alpha _{1}b+m(1-e^{-b\omega }) )} \biggl(\alpha _{2}+ \frac{b\alpha _{1}\alpha _{2}}{b+m(1-e^{-b\omega })} \biggr) \biggr] \\ &\quad {}\times\int _{0}^{t}I(\theta )\,d\theta +M(t)+\ln I(0). \end{aligned} $$

Note that

$$ d \biggl(S(t)+I(t)+me^{-b\omega } \int ^{t}_{t-\omega }S(s)\,ds \biggr)=b-bS(t)-m \bigl(1-e^{-b \omega }\bigr)S(t)-(b+\gamma )I(t),$$

then we have

$$\begin{aligned}& \frac{S(t)+I(t)+me^{-b\omega }\int ^{t}_{t-\omega }S(s)\,ds}{t}- \frac{S(0)+I(0)+me^{-b\omega }\int ^{0}_{-\omega }S(s)\,ds}{t} \\& \quad =b-\bigl(b+m\bigl(1-e^{-b\omega }\bigr)\bigr)\bigl\langle S(t)\bigr\rangle -(b+\gamma )\bigl\langle I(t) \bigr\rangle . \end{aligned}$$

Thus

4.5 $$ \bigl\langle S(t)\bigr\rangle = \frac{b}{b+m(1-e^{-b\omega })}- \frac{b+\gamma }{b+m(1-e^{-b\omega })}\bigl\langle I(t)\bigr\rangle - \frac{\phi _{1}(t)}{t}, $$

where

$$ \phi _{1}(t)= \frac{S(t)+I(t)+me^{-b\omega }\int ^{t}_{t-\omega }S(s)\,ds}{b+m(1-e^{-b\omega })}- \frac{S(0)+I(0)+me^{-b\omega }\int ^{0}_{-\omega }S(s)\,ds}{b+m(1-e^{-b\omega })}. $$

In view of Lemma 3.1, one can easily obtain that

$$ \lim_{t \to \infty }\frac{\phi _{1}(t)}{t}=0 \quad \mbox{a.s.},$$

so

4.6 $$ \bigl\langle S(t)\bigr\rangle = \frac{b}{b+m(1-e^{-b\omega })}- \frac{b+\gamma }{b+m(1-e^{-b\omega })}\bigl\langle I(t)\bigr\rangle , $$

by (4.4) and (4.6), we get

$$\begin{aligned} \ln I(t) \geq & \biggl[ \frac{\beta (b+m(1-e^{-b\omega }))}{b+\alpha _{1}b+m(1-e^{-b\omega })} \frac{b}{b+m(1-e^{-b\omega })}-(pb+\gamma ) \\ &{}-\frac{1}{2} \biggl( \frac{\sigma b}{b+\alpha _{1}b+m(1-e^{-b\omega })} \biggr)^{2} \biggr]t \\ &{}-\frac{\beta }{(b+\alpha _{1}b+m(1-e^{-b\omega }))} \biggl[(b+\gamma )+b \biggl(\alpha _{2}+ \frac{b\alpha _{1}\alpha _{2}}{b+m(1-e^{-b\omega })} \biggr) \biggr] \\ &{}\times\int ^{t}_{0}I(\theta )\,d\theta +\phi _{2}(t) \\ =&(pb+\gamma ) \bigl(R_{0}^{s}-1\bigr)t- \frac{\beta }{ (b+\alpha _{1}b+m(1-e^{-b\omega }) )} \\ &{}\times\biggl[(b+ \gamma )+b \biggl(\alpha _{2}+ \frac{b\alpha _{1}\alpha _{2}}{b+m(1-e^{-b\omega })} \biggr) \biggr] \int ^{t}_{0}I(\theta )\,d\theta +\phi _{2}(t), \end{aligned}$$

where $\phi _{2}(t)=M(t)+\ln I(0)- \frac{\beta }{(b+\alpha _{1}b+m(1-e^{-b\omega }))}\phi _{1}(t)$. Obviously, $\lim_{t \to \infty }\frac{\phi _{2}(t)}{t}=0$ a.s., by Lemma 4.1 and $R_{0}^{s} > 1$, we deduce that

$$ \liminf_{t \to \infty }\bigl\langle I(t)\bigr\rangle \geq \frac{(pb+\gamma )(R_{0}^{s}-1)(b+\alpha _{1}b+m(1-e^{-b\omega }))(b+m(1-e^{-b\omega }))}{\beta [(b+\gamma )(b+m(1-e^{-b\omega }))+\alpha _{2}b(b+m(1-e^{-b\omega })+\alpha _{1}\alpha _{2}b^{2})]}=I_{*}>0.$$

This is the required inequality (4.1), and from (4.5), we have

$$ \biggl\langle \frac{b}{b+m(1-e^{-b\omega })}-S(t) \biggr\rangle = \frac{b+\gamma }{b+m(1-e^{-b\omega })} \bigl\langle I(t)\bigr\rangle + \frac{\phi _{1}(t)}{t}. $$

Therefore,

$$\begin{aligned} \liminf_{t \to \infty } \biggl\langle \frac{b}{b+m(1-e^{-b\omega })}-S(t) \biggr\rangle &= \frac{b+\gamma }{b+m(1-e^{-b\omega })}\liminf_{t \to \infty }\bigl\langle I(t)\bigr\rangle \\ &\geq \frac{b+\gamma }{b+m(1-e^{-b\omega })}I_{*}>0. \end{aligned}$$

 □

## Simulations

In this section, we will use the Milstein method and the Euler–Maruyama method [40] to illustrate our results, and all the step sizes are 0.1 [41]. We take 50 realizations and use their average to plot such as $I(t)=\Sigma _{i=1}^{50}I_{i}(t)/50$, where the $I_{i}(t)$ represents the *i*th realization. We compare the threshold parameters of the deterministic model and stochastic model to explain the effect of white noise on the system. A typical example of vertical contagious and vaccine-related infectious diseases is hepatitis B. There are many studies on hepatitis B. In this part of numerical simulation, the value of parameters is taken from [42–45]. For the stochastic model (1.5), we consider the discrete equation:

$$ \textstyle\begin{cases} S_{k+1} =S_{k}+ [- \frac{\beta S_{k}I_{k}}{1+\alpha _{1}S_{k}+\alpha _{2}I _{k}+\alpha _{1}\alpha _{2}S_{k}I_{k}}-bS_{k}+pbI_{k}+b(1-I_{k})-mS_{k} \\ \hphantom{S_{k+1} ={}}{} +mS_{k-\omega }e^{-b\omega } ]\Delta t- \frac{\sigma S_{k}I_{k}}{1+\alpha _{1}S_{k}+\alpha _{2}I _{k}+\alpha _{1}\alpha _{2}S_{k}I_{k}} \sqrt{\Delta t}\xi _{k} \\ \hphantom{S_{k+1} ={}}{} -0.5\sigma ^{2} \frac{ S_{k}I_{k}}{1+\alpha _{1}S_{k}+\alpha _{2}I _{k}+\alpha _{1}\alpha _{2}S_{k}I_{k}}( \xi _{k}^{2}-1)\Delta t, \\ I_{k+1} =I_{k}+ [ \frac{\beta S_{k}I_{k}}{1+\alpha _{1}S_{k}+\alpha _{2}I _{k}+\alpha _{1}\alpha _{2}S_{k}I_{k}}- \gamma I_{k}-pbI_{k} ]\Delta t \\ \hphantom{I_{k+1} ={}}{} + \frac{\sigma S_{k}I_{k}}{1+\alpha _{1}S_{k}+\alpha _{2}I _{k}+\alpha _{1}\alpha _{2}S_{k}I_{k}} \sqrt{\Delta t}\xi _{k} \\ \hphantom{I_{k+1} ={}}{} +0.5\sigma ^{2} \frac{ S_{k}I_{k}}{1+\alpha _{1}S_{k}+\alpha _{2}I _{k}+\alpha _{1}\alpha _{2}S_{k}I_{k}}( \xi _{k}^{2}-1)\Delta t. \end{cases} $$

Here $\xi _{k}$ ($k= 1,2,\ldots$) is the $N(0,1)$-distributed independent Gaussian random variables. Now $\sigma (t)$ is the intensity of white noise and time increment $\Delta t>0$.

**Figure 2:**
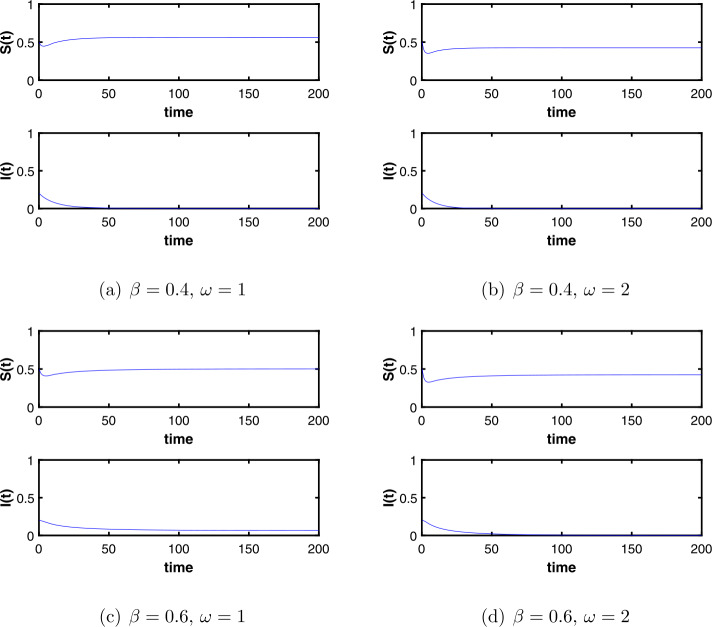
Dynamics of the deterministic system (1.2)

For the deterministic system (1.2), we choose the initial value $(S(0),I(0)) = (0.5,0.2)$ and the parameter values $\beta =0.4$, $b=0.3$, $p=0.1$, $m=0.9$, $\alpha _{1}=0.6$, $\alpha _{2}=0.1$, $\gamma =0.2$. We compare the two cases of $\omega =1$ and $\omega =2$ when $\beta =0.4$. By simple calculations, we get both of them $R_{0}^{2}<1$ and find that the *I* tend to 0, which means that the disease fades. In Fig. 2(a), (b), we see that the larger the value of the time delay *ω*, the faster the disease will fade. When $\beta =0.6$ and $\omega =1$, we get $R_{0}^{2}>1$, it shows that the disease becomes endemic, but when we increase the time delay to 2, we get $R_{0}^{2}<1$ and find that the disease is extinct from Fig. 2(c), (d). From the above analysis, it is found that the longer immune period, that is, time delay *ω*, the less likely the disease will break out.

**Figure 3:**
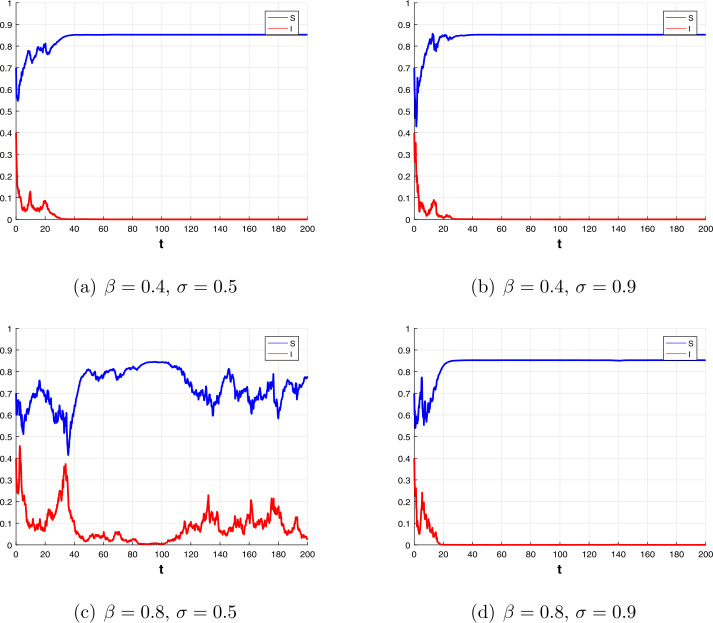
Dynamics of the stochastic system (1.5)

For the stochastic system (1.5), we first use the Milstein method [40] to illustrate our results, and we choose the initial value $(S(0),I(0)) = (0.7,0.4)$ and the parameter values $b=0.3$, $p=0.5$, $m=0.2$, $\alpha _{1}=0.6$, $\alpha _{2}=0.1$, $\gamma =0.2$, $\omega =1$. When $\beta =0.4$ and $\sigma =0.5$, we get $R_{0}^{s}=0.5310<1$ and $\sigma ^{2}\leq \frac{\beta (b+\alpha _{1}b+m(1-e^{-b\omega }))}{b}$; hence, the condition (b) of Theorem 3.1 is satisfied. When $\beta =0.4$ and $\sigma =0.9$, we get $\sigma ^{2}>\frac{\beta ^{2}}{2(pb+\gamma )}$, the condition (a) of Theorem 3.1 is satisfied. In Fig. 3(a), (b), the *I* both exponentially decays to zero, which indicates the extinction of the disease. Next, we let parameter $\beta =0.8$ and $\sigma =0.5$ and others are the same as above. In this case, we get $R_{0}^{s}=1.2712>1$, according to Theorem 4.1, the disease is persistent, see Fig. 3(c). As shown in Fig. 3(d), when *σ* increases to 0.9, the *I* also exponentially decays to zero, which indicates the extinction of the disease. The above results show that, to a certain extent, stochastic noise has an effect on infectious diseases, properly increasing noise intensity can reduce the spread of infectious diseases.

**Figure 4:**
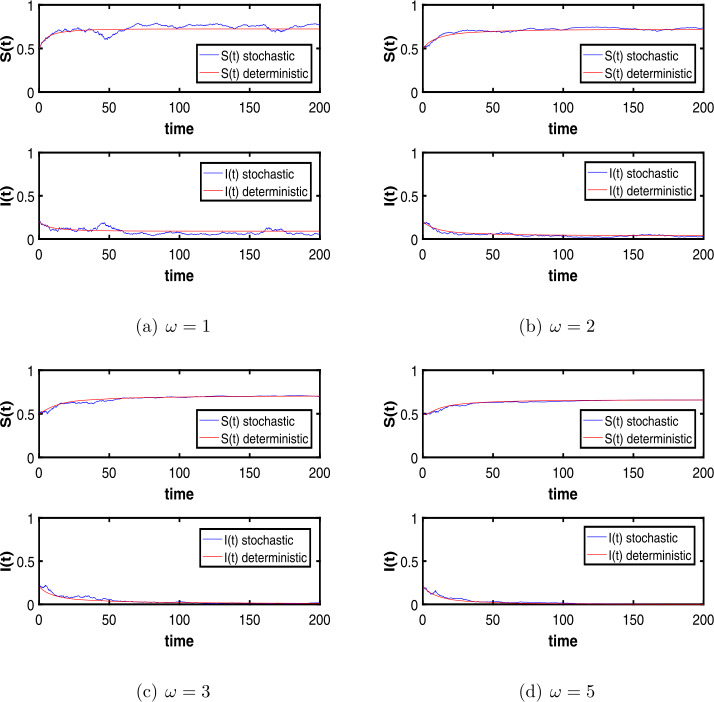
The effect of delay *ω* on dynamics of the stochastic system (1.5)

On the basic, we use Euler–Maruyama method and present the parameter values $\beta =0.7$, $b=0.3$, $p=0.5$, $m=0.2$, $\alpha _{1}=0.6$, $\alpha _{2}=0.1$, $\gamma =0.2$. Here we analyze the impact of time delay *ω* changes on infectious diseases when the noise intensity *σ* is little, i.e., $\sigma =0.2$. We find that when $\omega =1$, the disease is persistent and is shown in Fig. 4(a), when $\omega =2$ the disease is beginning to go extinct, until $\omega =3$ or $\omega =5$, the disease has become extinct, and they are shown in Fig. 4(b), (c), (d), respectively. Based on the above analysis, we can get that the time delay can contribute to the extinction of the disease, the larger the value of the time delay *ω*, the faster the disease will fade.

**Figure 5:**
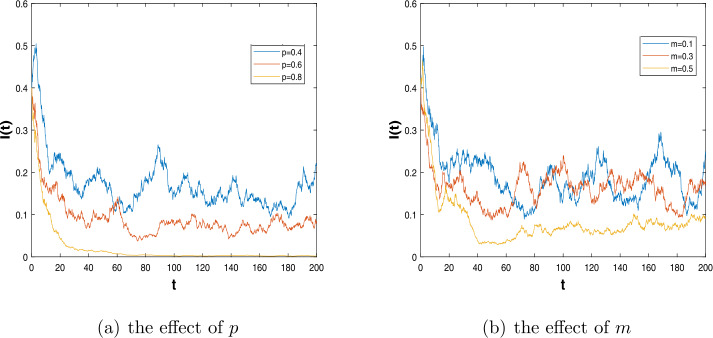
The effect of some parameters on dynamics of the stochastic system (1.5)

Next, we use the Milstein method [40] to analyze the influence of some parameters. We choose the initial value $(S(0),I(0)) = (0.5,0.4)$ and the parameter values $\beta =0.8$, $b=0.3$, $m=0.2$, $\alpha _{1}=0.6$, $\alpha _{2}=0.1$, $\gamma =0.2$, $\sigma =0.2$, $\omega =1$. Under these parameter values, we choose that in contrast, $p=0.4$, $p=0.6$ and $p=0.8$, see Fig. 5(a). The higher the p value, the more susceptible, the fewer people vertically infected; therefore, the disease will be quickly controlled. Then, we consider the proportion of successfully vaccinated population *m*, choose $p=0.5$, and other parameters do not change. Taking different values of *m*, we get that the bigger proportion of the successfully vaccinated population, the less infected, see Fig. 5(b).

**Figure 6:**
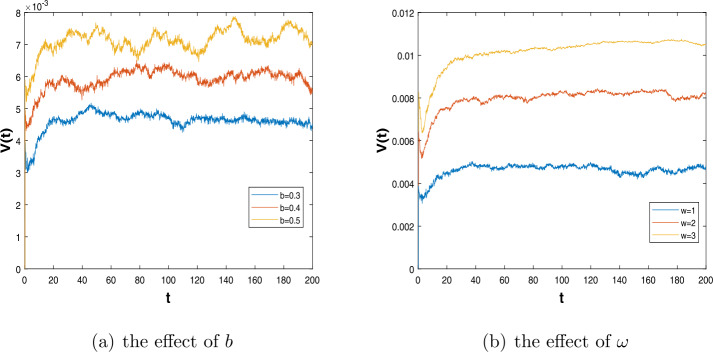
The immunity level

Lastly, we briefly describe the immunity level. We consider the form of successful immunization as $\frac{dV(t)}{dt}=mS(t)-mS(t-\omega )e^{-b\omega }$ and analyze how the immunity level changes as the time delay *ω* and the decay rate *b* change. We set $\beta =0.8$, $p=0.5$, $m=0.3$, $\alpha _{1}=0.6$, $\alpha _{2}=0.1$, $\gamma =0.2$, $\sigma =0.1$, and the initial value $(S(0),I(0)) = (0.5,0.4)$. Then, we can find when *b* is larger, the $V((t))$ is higher in Fig. 6(a). The higher the decay rate b, the smaller the probability of death due to disease, and then, the population size of successful vaccination is larger. On the other hand, we set $b=0.3$, and other parameters do not change. Then, in Fig. 6(b), we can find when *ω* is larger, the $V((t))$ is higher. The greater the time delay *ω*, the less likely it is to die from the disease, and then, the population size of successful vaccination is larger. This shows that the effective period of vaccine is the longer, the immunity level is higher.

## Conclusions

In this paper, we have analyzed a stochastic delayed $SIR$ epidemic model with vertical transmission and vaccination, the introduction of stochastic effect and time delay into deterministic models gives us a more realistic way of constructing epidemic model. In addition, we consider a specific functional response incidence rate. In this model, firstly, we have proved the global existence, positivity, and boundedness of the solution. In addition, we have shown that the disease fades when the white noise is large enough such that $\sigma ^{2}>\frac{\beta ^{2}}{2(pb+\gamma )}$. Moreover, when the noise is small, i.e., $\sigma ^{2}\leq \frac{\beta (b+\alpha _{1}b+m(1-e^{-b\omega }))}{b}$, the extinction of the disease can be determined by the value of $R_{0}^{s}$; if $R_{0}^{s}<1$, the disease fades. The persistence of the disease is determined by $R_{0}^{s}$, i.e., if $R_{0}^{s}>1$, the disease persists. Finally, we have simulated our theoretical result and have also found that when the white noise is small, the stochastic system is similar to the deterministic system, but when the white noise is large enough, the stochastic system will appear to be a different phenomenon. Large white noise can suppress the spread of disease. And the higher the p value, the higher the proportion of susceptible newborns and the fewer patients with vertical transmission. With the development of modern medical treatment, there will be more medical measures to block the vertical transmission of infectious diseases. In addition, the increase in vaccination rate *m* and time delay *ω* have some influence on the development progress of the disease, they can effectively suppress the occurrence of the disease under the right circumstances.

It is very meaningful to study the epidemic model with time delay caused by vaccination, and we can not ignore the influence of vaccination on some infectious diseases. Moreover, by studying the dynamic behavior of stochastic infectious disease system, we can reflect the actual phenomenon more accurately and reveal the influence of stochastic disturbance on infectious disease system, which is of great significance for the scientific prediction of disease development trend and epidemic prevention and control. It can help us offer some useful control strategies to regulate disease dynamics. In future work, we will consider the delayed $SIR$ model with different incidences to build more realistic models and analyze some other characteristics about them.

## Data Availability

The data we used for this research is from respective published articles that are cited.
